# Psychosis Early Intervention in Southwark – Gold Standard Prescribing vs Reality

**DOI:** 10.1192/bjo.2022.448

**Published:** 2022-06-20

**Authors:** Jenny Irvine, Xiaofei Fiona Huang, Yasamine Farahani-Englefield

**Affiliations:** South London and Maudsley NHS Foundation Trust, London, United Kingdom

## Abstract

**Aims:**

The RCPsych Standards for Early Intervention in Psychosis Services documents Gold Standard treatment, including: every service-user with psychosis is offered antipsychotic medication; if their illness does not respond to therapeutic dose of 2 different antipsychotics, they are offered clozapine. The Southwark Team for Early Psychosis (‘STEP’) in the South London and Maudsley Trust (SLaM) treats adults in their first 3 years of psychosis diagnoses. We aimed to compare prescribing practice in STEP to RCPsych Standards.

**Methods:**

STEP's caseload of 296 individuals was reviewed on 7th June 2021. Those excluded: inpatients/under Home Treatment Team; not yet assessed. Final number of outpatients assessed = 269. Data gathered: 1) Taking an AP? If taking an AP, the name and dose of AP. If not taking, trial discontinuation with medical advice or unsupervised refusal? 2) Remission status 3) Total number of AP trials. Uncertainties in categorisation were reviewed by the 2 other contributors.

**Results:**

In 269 outpatients on 7/6/21, 186(69%) were taking an antipsychotic (167:19 oral:depot), with a further 62(23%) recommended but declining. 21(8%) were not recommended, following change in diagnosis or resolution of psychotic symptoms.

7 service-users had down-titrated off AP with medical collaboration. All but 1 remained in remission. 35/47(74%) who discontinued AP independently had relapsed.

172 patients were reliably taking antipsychotic medication as prescribed. 56(32.5%) had ongoing psychotic symptoms (ranging from non-preoccupying residual delusions to distressing delusions/hallucinations). 4 symptomatic service-users were prescribed lower than BNF minimum effective doses.

Of those symptomatic and on hypothetically therapeutic doses (n = 52; median% of BNF Maximum Dose 50%; mean 54%), 26 were on their 1st AP, 26 on or beyond their 2nd AP. 8 service-users had ever trialled clozapine.

**Conclusion:**

Even in an experienced EI team for a highly psychiatrically morbid population, there remain gaps between best practice and actual prescribing.

Close to 1/3 of patients taking their prescription weren't in remission, almost all of whom had room to increase doses or trial an alternative medication. Clozapine is under-utilised in the treatment resistant group. For those who stopped AP, supervised tapering is a reasonable treatment option.

The next steps will be run as a quality improvement project addressing MDT and service-user barriers to assertive medication management:

- Trial methods to improve adherence (depot prescribing, psychoeducation, peer support)
- Encourage efficient up-titration and frequent MDT review of AP efficacy (empowering service-users self-management, care-coordinator opportunistic mental state assessments to trigger dose increase, medical review frequency)
- Identify and refer service-users suitable for clozapine

